# Use of Cosmetics in Pregnancy and Neurotoxicity: Can It Increase the Risk of Congenital Enteric Neuropathies?

**DOI:** 10.3390/biom14080984

**Published:** 2024-08-10

**Authors:** Kendra Jones, Lucas M. Wessel, Karl-Herbert Schäfer, María Ángeles Tapia-Laliena

**Affiliations:** 1 “Translational Medical Research” Master Program, Medical Faculty of Mannheim, University of Heidelberg, Theodor-Kutzer-Ufer 1-3, 68167 Mannheim, Germany; 2 Department of Pediatric Surgery, Medical Faculty of Mannheim, University of Heidelberg, Theodor-Kutzer-Ufer 1-3, 68167 Mannheim, Germany; 3 Working Group Enteric Nervous Systems (AGENS), University of Applied Sciences Kaiserslautern, Amerikastrasse 1, 66482 Kaiserslautern, Germany; karlherbert.schaefer@hs-kl.de

**Keywords:** Hirschsprung Disease (HSCR), congenital enteric neuropathies, Enteric Nervous System (ENS), pregnancy, neurotoxins

## Abstract

Pregnancy is a particularly vulnerable period for the growing fetus, when exposure to toxic agents, especially in the early phases, can decisively harm embryo development and compromise the future health of the newborn. The inclusion of various chemical substances in personal care products (PCPs) and cosmetic formulations can be associated with disruption and damage to the nervous system. Microplastics, benzophenones, parabens, phthalates and metals are among the most common chemical substances found in cosmetics that have been shown to induce neurotoxic mechanisms. Although cosmetic neurotoxin exposure is believed to be minimal, different exposure scenarios of cosmetics suggest that these neurotoxins remain a threat. Special attention should be paid to early exposure in the first weeks of gestation, when critical processes, like the migration and proliferation of the neural crest derived cells, start to form the ENS. Importantly, cosmetic neurotoxins can cross the placental barrier and affect the future embryo, but they are also secreted in breast milk, so babies remain exposed for longer periods, even after birth. In this review, we explore how neurotoxins contained in cosmetics and PCPs may have a role in the pathogenesis of various neurodevelopmental disorders and neurodegenerative diseases and, therefore, also in congenital enteric aganglionosis as well as in postnatal motility disorders. Understanding the mechanisms of these chemicals used in cosmetic formulations and their role in neurotoxicity is crucial to determining the safety of use for cosmetic products during pregnancy.

## 1. Introduction

### 1.1. Cosmetics Contain Neurotoxins

Cosmetics have been used on human bodies around the world since as early as 3000 BCE to cleanse, alter appearance, and enhance beauty [1]. In the modern cosmetic industry, the formulations of cosmetic products include an increasing number of chemical substances to improve product quality and effectiveness. However, many of these included substances can be characterized as neurotoxicants that have the capacity to damage and disrupt cellular activity in the brain and nervous system.

The diversity of toxic substances included in cosmetics is impressive: paraformaldehyde, benzalkonium chloride, micro/nanoplastics, benzophenones, parabens derivates, phthalates and trace heavy metals among many others. These common compounds have shown a variety of mechanisms that have the potential to induce cytotoxicity, genotoxicity, and, importantly here, neurotoxicity [2] (see Table 1).

Regulatory agencies like the Food and Drug Administration in U.S. (FDA, College Park, MD 20740-3835, U.S.) [3] and the Scientific Committee on Consumer Safety (SCCS) [4] in the European Union (European Commission, Brussels, Belgium), which also keeps a special database on cosmetic substances and ingredients called CosIng [5], perform strict safety monitoring and regulation of cosmetics to protect consumers from exposure.

Nevertheless, the presence of small concentrations of neurotoxins can still be detectable in the formulations due to contamination and different country regulations [6]. Also, exposure even to permissible concentrations of neurotoxins during pregnancy may involve a risk [7].

**Table 1:** Most common neurotoxic components in Cosmetics and Personal Care Products.

Compound	Types	Found in *	Mechanisms	References
Microplastics Nanoplastics	Polyethene (PE) Polypropylene (PP) Polyvinylchloride (PVC) Polystyrene (PS) Polylactic (PLA)	Exfoliating Mosturizes Toothpaste Lipsticks Nail polish Packages	Inflammation Neurotransmitters disruption ↑ Oxidative stress AChE Inhibition Cellular toxicity Lipid peroxidation Endocrine disruptors	[8] [8,9,10] [8,9,10,11,12] [8] [8,13] [9] [10]
Parabenes	Methylparaben (MtP) Butylparaben (BuP) Ethylparaben (EtP) Propyl paraben (PrP)	Shower gel Body cream Hair products Deodorant Fragances	Endocrine disruption Neurotoxicity ↑ Oxidative stress Microbiote alterations	[13,14] [14,15,16,17] [17] [18]
Benzophenones	BP-1 BP-2 Oxybenzone-3/BP-3	Sun blockers Fragances	Neuronal migration MAPK/ERK signaling AChE Inhibition	[19] [20] [21]
	BP-4		Neurotransmitters disruption	[22]
Phthalates	Di-ethyl-phthalate (DEP)Di-n-butyl phthalate (DBP) Dimethyl-phthalate (DMP)	Eyeshadows Fragances Nail polish Moisturizers	Endocrine disruption ↑ Oxidative stress AChE Inhibition Microbiote alterations	[23,24,25] [8] [8] [26] [24,27]
		Hair products	Cellular apoptosis	
Metals Trace metals	Lead (Ld) Aluminium (Al) Cadmium (Cd) Nickel (Ni) Arsenic (As) Mercury (Hg) Manganese (Mn) Titanium dioxide (TiO2) Chromium (Cr) Iron (Fe) Copper (Cu) Cobalt (Co)	Lipsticks Eyeshadows Lotions Powders Additives Mascaras Foundations Sun blockers Toothpaste Eye products Additives	↑ Oxidative stress AChE Inhibition Autophagia Apoptosis Microbiota alterations Blocking Ca^2+^/K^+^ channels Neurotransmitters disruption Endocrine disruption	[8,28,29,30] [8,31] [28,32] [33,34] [26] [35] [36] [37]

* (list of mainly products containing them, among many others). ↑: increase of; AChE: Acetylcholinesterase.

Prior to the 1960s, the harmful effects of cosmetics were unknown. It was believed that cosmetics would remain on the surface of the body and exposure to the chemical compounds from the formulations was limited due to the protective barrier function of skin [38]. However, given the multiple exposure scenarios of cosmetics, systemic exposure of toxins following cosmetic application has been proven possible. Systemic exposure can happen through percutaneous penetration of products applied to the skin, inhalation of spray products, or ingestion of products applied around the oral cavity. The degree of systemic exposure is unclear and can depend on several factors, including the concentration of the formulation, application area, amount and frequency of application, and duration of product use [37]. The complete formulation of the cosmetic is also important to consider as the inclusion of surfactants and co-solvents can help enhance skin penetration and dermal absorption of topically applied cosmetics [39]. This variety of factors makes it difficult to determine the concentration extent to which humans may be exposed to the toxins.

Given that most cosmetic products are applied topically, dermal absorption is the most common route of exposure. Once the toxin passes through the epidermis it reaches the blood stream where it can then travel everywhere. Toxins have been detected in human fluids and several tissues, which demonstrates their dissemination through the human body [40].

### 1.2. Neurotoxins Pass the Placental Barrier and Accumulate in Breast Milk

Common neurotoxic ingredients in cosmetics have been detected in a variety of human tissues, including in maternal blood and urine, the placenta, or even the umbilical cord (see Table 2).

First, neurotoxicity can damage the Blood Brain Barrier (BBB), affecting the barrier structure or disrupting the barrier’s regulatory functions [41]. In addition to crossing the BBB, many neurotoxins, such as parabens and ultraviolet filters [42], can cross the placental barrier, making prenatal exposure a concern. Prenatal exposure to toxins may be especially harmful given that the developing brain is a more susceptible target to toxicity as neurons proliferate, migrate, and forge important synaptic connections to create an optimum adult brain structure [43].

Similarly, neurotoxic metal concentrations have been measured in placenta tissue samples and were demonstrated to affect birth outcome, where some metals adversely affected fetal growth [44]. Metals have been associated to derived neurodevelopmental and cognitive problems in children [45]. Benzophenone-3 and methylparaben exposure also correlated to lower birthweight in a large cohort analysis of American children [46].

Unfortunately, the toxicity of these chemicals is not limited to pregnancy: there is evidence of the presence and concentration of bisphenols, parabens (PBs), and benzophenones (BPs) in human milk [47], which implies that babies are exposed even after the gestational period.

**Table 2:** Detected mother/child transmission.

Detected in	Compound	References
Placenta barrier	Microplastics Parabens Benzophenones Metals	[48,49,50]
		[42] [42]
		[44]
Breast milk	Microplastics Bisphenol Parabens Benzophenones	[47] [47] [47,51] [47]
Maternal Urine/Blood	Benzophenones Parabens	[19,52] [14]
Umbilical cord	Benzophenones	[52]
Intestine	Microplastics	[10,49,53,54]
	Parabens	[40]
	Phthalates Metals	[26,40] [26]
Brain	Microplastics	[8,10,49]
	Parabens Benzophenones Phthalates Metals	[17] [22] [26] [26]

## 2. Prenatal Exposure and Risk of Enteric Neuropathies

Maternal exposure to environmental toxins represents a major risk for the health of the newborn (see Table 3). Pregnant women are exposed daily (even unconsciously) to many toxicants through air, water, food, and drugs, but also cosmetics. Smoking and alcohol are also well-known factors that severely compromise development. The toxicity of some compounds has been well-known by society for years; thus, it is easy to label them as negative and dangerous for pregnant women, as in, for instance, ethanol consumption [55]. Other compounds are recognized as adverse but not so easy to avoid, like the exposure to pesticides such as glyphosate [7,56] or microplastics [48,57]. However, the harmfulness of many compounds is not so obvious for future mothers, although they have already been related to neuronal damage, like commonly used drugs such as ibuprofen [58], anti-depressants [59,60], or cosmetics ingredients like oxybenzones (BP-3) [22,61].

Not only is the consumption of cosmetics by pregnant women quite frequent [62,63], but, also, a recent study collecting questionnaires from pregnant women reported that their risk perception seems to be low [64].

Enteric neuropathies are characterized by their impairment of the innervation of the gastrointestinal tract (GIT). There are various clinical phenotypes, depending on the location and degree of the innervation absence, such as esophageal achalasia, gastroparesis, Hirschsprung disease, or intestinal pseudo-obstruction syndromes [65,66,67]. Despite these gastrointestinal disorders presenting high long-term morbidity and mortality, current therapeutic approaches are mainly supportive, rather than curative [65,66].

One of the most studied aganglionic diseases is Hirschsprung Disease (HRSC, incidence 1/5000), a congenital gastrointestinal condition in which the intrinsic gastrointestinal innervation of the colon is absent or severely affected due to a failure of enteric neural crest cell migration during early embryogenesis (from 5 to 12 weeks) [68,69]. The regulation of this process is critical, and many different genes and proteins are involved in both migratory and colonization processes [70]. Currently, the only treatment is surgical removal of the aganglionic bowel. Nevertheless, patients suffer complications such as toxic megacolon and enterocolitis, leading to poor long-term outcomes [71]. Although some genes have been identified, the condition is not fully explained by the genetic load [72,73] and many non-genetic factors also affect the development of the enteric nervous system (ENS) and, so, impact the risk [68,74]. Altogether, these point to an environmental factor as a disease co-cause or trigger, suggesting that some cases of HSCR might be preventable [75].

While HSCR is a severe disease with a huge impact on quality of life, there might also be minor impacts on ENS development that do not lead to obvious damage but rather to mild clinical symptoms such as continuous obstipation or else.

Consequently, maternal exposure to neurotoxins during early pregnancy may disturb the proper migration of the enteric neural crest cells, which is essential for a correct innervation of the whole GIT [69], and, so, represent a risk for congenital aganglionosis or, in milder cases, for the development of less severe degrees of motility disorders or milder symptoms in children [76,77]. Considering that ENS development continues for months after birth [69], further exposure through neurotoxins contained in breast milk may also impact appropriate GIT innervation in babies.

Cosmetic toxicants have been pointed to act as disruptors of the gut microbiota, namely to gastrointestinal dysbiosis, which can later cause neurotoxicity [40]. Indeed, alterations in the gut microbiome in combination with individual genetics could potentially transform the Enteric Nervous System (ENS), central nervous system, and immune system, impair barrier function, and contribute to various disorders such as irritable bowel syndrome, inflammatory bowel disease, or neurodegeneration and mental issues [26,78].

Though the fetal GIT microbiota has usually been considered absent and colonized after birth, recent studies have detected microorganisms in amniotic fluid, fetal membranes, umbilical cords, placentas, and meconium [79]. Indeed, the fetal GIT seems to be first colonized by swallowing amniotic fluid bacteria [79]. These observations suggest that the maternal intestinal microbiota is possibly transmitted to the fetus and can influence fetal development [79,80]. There are two main proposed pathways on how the gestational intestinal microbiota could exert significant effects on fetal development, first by colonization of fetal tissue by the maternal gut microbiome; second, by the placental transport to the fetus of maternal microbiota-derived metabolites and compounds [80]. This supports our hypothesis that neurotoxicants that affect the maternal microbiota during pregnancy may also impact fetal neuronal development. In addition, the maternal microbiota contained in breast milk could also influence the last stages of ENS development in the newborn further on [69].

**Table 3:** Neurotoxic compounds and associated embryo/development complications.

Compound	Impairment	Complications	References
Microplastics	Endocrine Disruption Neurotoxicity Inflammation Oxidative stress	Neurodevelopmental Cognitive Behavioral Microbiota dysbiosis Parkinson-like Disease (PLD)	[10,54,81,82,83] [10,54,81,82,83] [10] [54,81,82,83] [10]
Benzophenones	Cellular migration Neurotoxicity	Hirschsprung Disease (HSCR) Cognitive	[19] [84]
Parabenes	Endocrine Disruption	Children overweight	[14]
	Oxidative stress Mitochondrial dysfunction Neuroinflammation	Autism Spectrum Disorder (ASD) Cognitive	[17] [84,85]
Phthalates	Endocrine disruption	Attention-deficit Hyperactivity Disorder (ADHD)- behavioral profile	[86]
	Apoptosis Oxidative stress Microbiota dysbiosis	Anxiety Mental disorders	[87] [26]
Metals	Autophagia Apoptosis Oxidative stress Microbiota dysbiosis	Memory, motor skills Parkinson Disease (PD), Alzheimer Disease AD	[28] [28,88]
		Cognitive impairment Amyotrophic Lateral Sclerosis (ALS) Mental disorders	[37,89] [29,90,91] [26]

## 3. Overview of Main Neurotoxins Contained in Cosmetics and PCPs

### 3.1. Microplastics and Nanoparticles

Microplastics (5 mm at 0.1 µm) and nanoplastics (<0.1 µm) had been extensively used as ingredients in personal care products (PCPs) and cosmetics [13,92,93]. Furthermore, microplastics have emerged as a new, huge, and ubiquitous health and environmental problem all over the world [49]. They also pass into the food chain almost inevitably from food packaging, cooking pots, and plastic bottles [53,94]. As a result, we are eating and drinking them every day and they accumulate in our intestines and organs [53,54,94].

The harmful effects of microplastics can be even worse, because they act as carriers of other dangerous chemicals such us heavy metals (Al, Cd, Co, Cr, Cu, Hg, Mn and Pb), polycyclic aromatic hydrocarbons (PAHs), polychlorinated biphenyls (PCBs), pesticides, and persistent organic pollutants (POPs). All these can bioaccumulate and impair oxidative stress and cellular toxicity [13].

Aside from this “environmental corona” of pollutants, microplastic particles also glue a “biological corona” of proteins and biomolecules on their surface when they are exposed to biological fluids. The composition of the corona defines their ability to enter the BBB and their overall toxicity, since this surface layer regulates their thermodynamics for diffusion into the membrane, and it can allow for the plastic to be absorbed into the membrane and cross over to the neural tissue [95]. Microplastics can also cross the BBB by disrupting the tight junctions [10,96].

Therefore, microplastics have been reported to cross the BBB, induce inflammation and oxidative stress, disrupt neurotransmitters and, so, lead to neurotoxicity [10,49], brain damage, and impaired neuronal development in different animal models [8,9,10,11,12]. Moreover, microplastic effects can contribute to the development of other diseases such as central nervous system inflammation and Parkinson’s-like (PLD) neurodegenerative disorders [10].

Regarding the epithelial barrier, through the skin, the corneous layer is limited to particles lower than 100 nm, so nanoplastic absorption is more probable than microplastics by this way [49].

Ingested microplastic particles smaller than 2.5 µm can cross the GIT through endocytosis by M cells at Peyer’s patches. M cells transport particles from the intestinal lumen to the mucosal lymphoid tissues or through paracellular persorption, which involves mechanical kneading of solid particles through gaps located in the single-layer epithelium at the villus tips of the GIT and into the circulatory system [49]. In nanoparticles, endocytosis and phagocytosis have also been described as intestinal uptake mechanisms [49,97].

In addition, microplastics have been found to accumulate in the digestive system, where they damage the gut barrier and cause intestinal problems like inflammation and microbiota dysbiosis [54,81,82,83], which can induce neurotoxicity and neurodegeneration [26,78].

Microplastics contained in breast milk [47] will reach the newborn GIT, where they may not only induce mucosal damage and inflammation and, therefore, increase intestinal permeability (potentially increasing their access to their ENS), but also affect the microbiota and reach the blood circulation [98].

Furthermore, maternal exposure to microplastics like polystyrene has already been proved to cause metabolic disorders in the offspring [57], and microplastic nanoparticles are able to cross the placental barrier and also to accumulate in it [48,49,50].

Altogether, microplastics can nave neurotoxic activity, affect the nervous system, and reach the fetus, so they are good candidates to be disruptors of the development of the enteric nervous system and intestinal innervation during fetal development.

### 3.2. Parabens

Parabens (PBs) are commonly used in the cosmetic industry as preservatives. Mainly, PBs are already reported to be important hormonal disruptors and to interact with estrogen, androgen, and thyroid receptors [13].

Given this, we should not forget that pregnancy is a period particularly vulnerable to the potential risks of the endocrine disruptors. In a study that linked maternal paraben exposure to childhood obesity, maternal urine samples at the 34th week of gestation contained detectable paraben concentrations. Of note, mothers that used paraben-containing cosmetic leave-on products on a daily basis had significantly higher urinary paraben concentrations [14]. However, exposure to PBs continues immediately after birth: PBs can also be found in breastmilk [47] and in processed infant food products, like milk-based infant formula and cereal-based complementary foods [51].

Several studies have reported PB neurotoxicity. For instance, by interacting with the estrogen receptor, butylparaben (BuP) can drive apoptosis in primary cortical neurons in vitro and, so, was identified as a potential contributor to neurodegeneration in the brain [99]. In rat models, BuP displayed several neurotoxic activities, such as increased oxidative stress, decreased reduced glutathione levels and elevated oxidized glutathione, mitochondrial dysfunction, and neuroinflammation, among others [17]. Other studies using zebrafish embryos demonstrated the neurotoxicity of methyl- (MtP), ethyl- (EtP), and propyl-parabens (PrP) [100]. Also, MtP and BuP produced neurobehavioral toxicity in adult samples of zebrafish [15,16]. In animal models, prolonged exposure to PBs provokes microbiota alterations [26], which can cause ENS toxicity [78].

Concerning children, PBs are associated to attention and neurocognitive problems in small children [84,85] and were also linked to sensorineural hearing loss [101]. In addition, parabens have been related to autism spectrum disorders (ASDs) [17].

Hence, endocrine disruptors like PBs can be detected in high concentrations in expecting mothers and are neurotoxic and can induce neurodevelopmental problems in children.

### 3.3. Benzophenones

In recent years, benzophenones, mainly ingredients of sunscreen cosmetics (see Box 1), have also been reported as neurotoxicants. Of them, Benzophenone-3 (BP-3, also known as Oxybenzone 3) is one of the most widely used UV filters, showing weak estrogen and strong anti-androgenic effects [19]. Also, it is small enough to pass through skin and placental barriers, and it has been detected in the urine/blood of pregnant women as well as in fetal and umbilical cord blood [52]. In addition, BP-3 has good permeability through the BBB [22].

Studies using zebrafish models have shown that BP-3 exposure leads to altered enzymatic activity (Glutathione S-transferase -GST, catalase -CAT, and acetylcholinesterase -AChE), neurotoxicity, and behavioral alterations [21]. Notably, BP-3 treatment in zebrafish resulted in enteric neuron loss and impairment of ENS development by inhibiting the MAPK/ERK signaling pathway [20]. Also, prenatal exposure to BP-3 dysregulates expression of neurogenesis- and neurotransmitter-related genes in the offspring [22]. In pregnant mice, exposure to BP-3 affected placental function and morphology [102].

Regarding benzophenone exposure and its consequences for human health, prenatal exposure to benzophenones has been related to neurocognitive problems in children [84].

Furthermore, BP-3 has been proven to disrupt neuronal migration in in vitro neuronal cultures [19]. Maternal exposure to BP-3 was also epidemiologically associated to a higher HSCR incidence in children, as higher urine concentrations correlated with higher HSCR [19]. Aside from that, under normal conditions BP-3 can travel to the maternal blood reaching the fetus at high enough levels to inhibit migration of neural crest cells during critical embryonic development [19,52].

Therefore, the use of benzophenones during pregnancy does increase the risk of enteric aganglionosis in children.

Box 1 **Sunscreen cosmetics show increasing risk of neurotoxicity.**

  Many metals are used as UV filters, and they have been found to be an increasing toxicity risk when reacting with UV rays. Titanium dioxide, commonly used in sunscreen, causes the formation of hydroxyl radicals when exposed to UV and leads to oxidation damage to DNA. This increases the risk for induced neurotoxicity if it is absorbed through the skin [103]. In the context of sunburn, it should also be recognized that damaged sunburnt skin may be more susceptible to chemical absorption [104]. In conjunction with both factors, nanometals have also become incorporated into cosmetic formulations, specifically TiO_2_ in sunscreen, to achieve higher quality products. This poses a further threat in inducing neurotoxicity. While there are unknowns about the impact nanoparticles have on the body, the small size of these substances suggest that they allow for deeper skin penetration [105]. In addition, oxybenzones like BP-3, usually present in sunblock, have already been associated with congenital enteropathies like Hirschsrpung Disease [19,20]. Given these factors together, it is important that the neurotoxic effects of sunscreen products be considered more closely in the future.

### 3.4. Phthalates

Phthalates are a family of chemicals used as plasticizers and solvents in various cosmetic products including eyeshadows, perfume, nail polish, moisturizers, and more [106]. Specifically, low molecular weight phthalates, Di-ethyl-phthalate (DEP), Di-n-butyl phthalate (DBP), and Dimethyl-phthalate (DMP), have a history of being used in cosmetic formulas [107]. There are several neurotoxic mechanisms of action that have been associated with phthalates including endocrine disruption, oxidative stress, and cellular apoptosis.

Thyroid hormone regulation plays a crucial role in brain development, and it can be disrupted by phthalate exposure. DBP exposure can lead to thyroid receptor T3 inhibition and thyroid hormone disruption [108,109]. DBP may also disrupt estrogen receptor signaling [110]. Like thyroid hormone signaling, estrogen also has effects on brain neurogenesis and neuroplasticity [110].

In addition to hormone signaling disruptions, phthalates have also been shown to increase oxidative stress by different mechanisms. DBP has proven to increase Reactive Oxygen Species (ROS) levels in vivo [23] and in vitro [24], as well as to increase of malondialdehyde (MDA) and nitric oxide (NO) levels in rats [25].

Induced apoptosis is another supported neurotoxic mechanism of phthalates [24]. In a rat model, prenatal DBP exposure resulted in caspase-3 activation as well as hippocampal neuron loss and structural alterations [33].

Numerous studies have linked phthalate exposure to human neurodevelopment disorders, with microbiota dysbiosis being the probable nexus in-between [26]. For instance, Attention Deficit Hyperactivity Disorder (ADHD) behaviors such as inattention and impulsivity have shown to have a positive correlation with DBP phthalate metabolite concentration found in urine of school-age (8–11 years) human subjects [111]. In addition, Engel et al. evaluated various parent-rated behavior differences of children ages 4 to 9 in relation to prenatal phthalate metabolite concentration in urine. They found mothers with higher metabolite concentrations had children with poorer scores associated with aggression, conduct, attention, externalizing problems, and, overall, all behavior symptom index summary scores. These results are also suggestive of the ADHD behavioral profile [112]. Animal behavior models have also agreed with these findings showing increases spontaneous motor activity in rats following DBP exposure, indicating the hyper-activity behavior that is common in ADHD [86]. Anxiety-like behaviors have also been demonstrated to increase following DBP exposure in mice using an elevated plus maze and open-field test [4]. While these studies have agreed with the implications that phthalate neurotoxic mechanisms may lead to developmental disorders, there are other developmental disorders such as Autism Spectrum Disorder (ASD) that have shown contradictory results in association with phthalate exposure [27].

Given that phthalates are verified neurotoxicants with proven neuronal impairment consequences, there are good reasons to analyze their neurotoxic effects during ENS development.

### 3.5. Metals

Another dangerous neurotoxic component included in cosmetic formulations are metals, including both heavy metals like lead (Pb), cadmium (Cd), nickel (Ni), arsenic (As), mercury (Hg), and manganese (Mn) and trace metals like chromium (Cr), iron (Fe), copper (Cu), and cobalt (Co). These are found in different cosmetics at various concentrations often used for pigmentation in makeup products or as UV filters in sunscreen (see Box 1) [6]. Human exposure to metals has been shown to induce neurotoxicity through different mechanisms such as autophagy, synaptic transmission, and oxidative stress. Autophagy occurring in the brain is a mechanism of neurodegenerative disorders such as Parkinson’s disease (PD) and Alzheimer’s disease (AD). In a rat model, intrastriatal injection of manganese showed compensatory activation of autophagy in the short term [28]. Similarly, an overload of iron increased autophagic activity in in vitro neuronal cultures [32].

Metals may also induce neurotoxicity through disruption in synaptic transmission between neurons as they are highly involved in ion channels, neurotransmitter release, and neurotransmitter receptors. For example, in hippocampal neuron cultures, lead exposure has been shown to increase the release of both glutamate and GABA neurotransmitters independently of calcium. This indicates that a metal like lead has targets present at presynaptic terminals that can alter neurotransmitter release [36]. More importantly, lead has also been found to be capable of blocking both calcium channels and potassium channels that are critical in helping regulate neuron signaling [35]. In addition to signaling, metals may also affect neurotransmitter metabolism. Pohanka et al. showed that copper, aluminum, iron, and calcium inhibit acetylcholinesterase (AChE), an enzyme that hydrolyzes acetylcholine, which leads to an effect in cholinergic neurotransmission [31].

Like phthalates, oxidative stress (see Box 2) is a well-known mechanism of metal toxicity. This is due to the fact that metals are redox-active, accepting or donating electrons, and can undergo redox cycling reactions creating ROS and Reactive Nitrogen Species (RNS) to result in oxidative stress [30]. Most ROS production has been found to be generated by the reaction of oxygen with copper and iron [29]. Metal production of ROS can play a key role in the coordination of signaling, as shown by Hidalgo et al. who demonstrated iron can generate calcium signals with ROS-mediated stimulation. However, in the presence of too much iron, calcium signaling may become excessive, leading to neuronal cell death [34].

Overall, increased exposure to metals may result in a defective metal homeostasis, which can contribute to neurodegenerative diseases (see Box 2). In the pathology of Amyotrophic Lateral Sclerosis (ALS), it is hypothesized that increased copper levels may result in defective redox chemistry and subsequent ROS generation in ALS patients [90]. This is supported by studies that have shown copper chelators can inhibit the progression of ALS in mouse models [29]. Aside from ALS, AD and PD patients also showed increased levels of copper and zinc. However, it is unclear whether these metals play a role in the onset of the disease or in its progression [88]. In addition, one ALS study found higher metal concentrations in CSF than in blood, indicating mechanisms of inward transport of metals [91].

In addition to neurodegenerative diseases, metals may also influence neurodevelopment. Dual exposure of lead and mercury during late pregnancy has shown to have negative effects on the mental development index evaluating problem solving, memory, classification, and motor skills of infants at 6 months [92]. Like phthalates, heavy metals alter the gut microbiota, which induces mental health problems [26].

All these toxicity mechanisms and associations to neuronal disorders and gastrointestinal dysbiosis, support metal neurotoxicity as a probable disruptor of the enteric neuronal system development.

Box 2 **Oxidative stress plays a key role in cosmetic induced neurotoxicity and may contribute to neuro-degenerative diseases.**

  In both phthalates and metals, oxidative stress appears in many models as a key player of induced neurotoxicity in cosmetics. Oxidative stress is characterized as defective redox homeostasis due to the accumulation of Reactive Oxygen Species (ROS) and Reactive Nitrogen Species (RNS) or a decrease in antioxidant enzymes [113]. Oxidative stress is involved with other effects that could induce neurotoxicity, including DNA damage, oxidizing proteins, induced lipid peroxidation, and cell apoptosis [114]. Given the high consumption of oxygen in the central nervous system, the brain is vulnerable to oxidative stress injury [115,116]. In addition, the brain has been shown to have lower antioxidant defense capabilities than other tissues [25].

  Oxidative stress is known to have an effect in neurogenerative diseases such as Alzheimer’s disease (AD), Parkinson’s disease (PD), and Amyotrophic Later Sclerosis (ALS). It is unclear whether oxidative stress is an initiator of the disease pathologies or simply a consequence of brain degeneration. However, it is hypothesized that accumulative oxidative damage over time accounts for late life onset of these diseases.

## 4. Conclusions

There are many cosmetic products containing toxic components like microplastics, parabens, benzophenones, phthalates, and metals that are used by pregnant women and impose neurotoxic threats to the human body. This may be associated with the fact that the extent of exposure is believed to be at too low of a concentration to have an effect or because users care more about the quality of the product than its health effects. However, given the different use and exposure scenarios of cosmetics, it is not clear which toxin concentrations are dangerous and what their effect is on the human body [89].

Despite literature that outlines the potential neurotoxicity of these substances, found in personal care products and cosmetics, they continued to be used in formulations and ignored by consumers. Together, this means that there is work that needs to be done to understand a proper exposure model of cosmetic neurotoxins, as well as to understand the direct causal relationships between the mechanisms of the chemical and its neurotoxic effect.

However, special attention should be paid to the consumption and composition of cosmetic and personal care products during pregnancy. Exposure to neurotoxins contained in these formulations during gestation could impair enteric neuronal development (and the complete neural colonization of the whole gut), as well as compromising the neuroimmunological interaction. Thus, it may cause congenital aganglionosis, as well as postnatal motility disorders as a consequence. Here we would like to inspire a more intense investigation concerning the specific effects of the above-mentioned compounds to create a catalogue of potentially harmful substances for the ENS.

Finally, the urgent need for eradicating these neurotoxic compounds from all formulations is emerging.
